# C3G Protein, a New Player in Glioblastoma

**DOI:** 10.3390/ijms221810018

**Published:** 2021-09-16

**Authors:** Sara Manzano, Alvaro Gutierrez-Uzquiza, Paloma Bragado, Angel M Cuesta, Carmen Guerrero, Almudena Porras

**Affiliations:** 1 Departamento de Bioquímica y Biología Molecular, Facultad de Farmacia, Universidad Complutense de Madrid (UCM), 28040 Madrid, Spain; sara.manzano@biodonostia.org (S.M.); alguuz@ucm.es (A.G.-U.); pbragado@ucm.es (P.B.); angcuest@ucm.es (A.M.C.); 2 Instituto de Investigación Sanitaria del Hospital Clínico San Carlos (IdISSC), 28040 Madrid, Spain; 3 Instituto de Investigación Sanitaria (IIS) Biodonostia, 20014 San Sebastián/Donosti, Spain; 4 Instituto de Biología Molecular y Celular del Cáncer (IMBCC), Universidad de Salamanca-(CSIC), 37007 Salamanca, Spain; 5 Instituto de Investigación Biomédica de Salamanca (IBSAL), 37007 Salamanca, Spain; 6 Departamento de Medicina, Universidad de Salamanca, 37007 Salamanca, Spain

**Keywords:** C3G, glioblastoma, Rap

## Abstract

C3G (RAPGEF1) is a guanine nucleotide exchange factor (GEF) for GTPases from the Ras superfamily, mainly Rap1, although it also acts through GEF-independent mechanisms. C3G regulates several cellular functions. It is expressed at relatively high levels in specific brain areas, playing important roles during embryonic development. Recent studies have uncovered different roles for C3G in cancer that are likely to depend on cell context, tumour type, and stage. However, its role in brain tumours remained unknown until very recently. We found that C3G expression is downregulated in GBM, which promotes the acquisition of a more mesenchymal phenotype, enhancing migration and invasion, but not proliferation. ERKs hyperactivation, likely induced by FGFR1, is responsible for this pro-invasive effect detected in *C3G* silenced cells. Other RTKs (Receptor Tyrosine Kinases) are also dysregulated and could also contribute to C3G effects. However, it remains undetermined whether Rap1 is a mediator of C3G actions in GBM. Various Rap1 isoforms can promote proliferation and invasion in GBM cells, while C3G inhibits migration/invasion. Therefore, other RapGEFs could play a major role regulating Rap1 activity in these tumours. Based on the information available, C3G could represent a new biomarker for GBM diagnosis, prognosis, and personalised treatment of patients in combination with other GBM molecular markers. The quantification of C3G levels in circulating tumour cells (CTCs) in the cerebrospinal liquid and/or circulating fluids might be a useful tool to improve GBM patient treatment and survival.

## 1. Introduction

### 1.1. Glioblastoma: Generalities

GBMs (glioblastomas), or grade IV astrocytoma/gliomas [1], are the most frequent and aggressive type of cancer originated in the CNS (central nervous system) (four new diagnoses per 100,000 population and 12–14 months of median survival rate in patients) [2]. Females present a lower incidence and slightly better prognosis than males [3]. First-line treatment includes surgical resection of the main tumour mass followed by adjuvant radiotherapy, combined with the alkylating agent, TMZ (temozolomide) [4]. In 2016, the WHO subclassified GBMs in two main subtypes: GBM-IDH (isocitrate dehydrogenase)-wildtype (almost 90% of cases) and GBM-IDH-mutant (around 10%) [5]. Recently, a novel WHO classification considered GBMs as adult-type diffuse gliomas, distinguishing GBM-IDH-wildtype from the astrocytoma-IDH-mutant [6]. At the molecular level, GBMs have also been categorised into different subtypes, being the four subtypes, neural, proneural, classical, and mesenchymal, the most widely used. This classification reflects altered signalling pathways, microenvironment composition, treatment responses, and prognosis [7,8,9,10,11].

Despite GBM intra- and inter-patient heterogeneity, a number of alterations are directly associated with this type of tumour, leading to the definition of diverse molecular signatures. RTKs (receptor tyrosine-kinases) and their downstream pathways are altered in almost 90% GBMs. The most commonly altered RTK is EGFR (mutated or amplified in 50–60% of cases), followed by PDGFRα (amplified in 13% of cases), ErbB2 (mutated in 8% of cases), MET (amplified in 2–4% of cases), and FGFR1 (amplified in 3.2% of cases) [12,13]. In addition, intracellular signalling pathways are frequently altered in GBM. For example, the Ras pathway is usually over-activated through mutation or deletion of NF1 (a Ras GAP (GTPase activating protein)). Other components of intracellular signalling cascades such as PTEN, PI3K, p53, or pRB are also recurrently altered in GBM [12,13].

GBM patients have poor prognosis due to therapy resistance, secondary foci formation, and tumour regrowth/recurrence. Two tightly controlled cell capacities have been associated with them: stemness [14,15] and dissemination [16].

Regarding the first one, CSCs (cancer stem cells) are defined as a small subpopulation of cancer cells with stem and initiating capabilities, responsible for tumour onset and therapy resistance. CSCs present in GBM, known as GSCs (GBM stem cells), display self-renewal capacity, ability to differentiate, and tumour initiating properties [15]. They express stem-related markers (SOX2, Mushashi, Bmi, Nestin, CD133, or CD44) and maintain basal proliferation and anti-apoptotic mechanisms without decreasing their stem-like phenotype [14].

Concerning dissemination, it is important to point out that GBMs rarely generate extra-neural metastases, but they have a high capacity to infiltrate the surrounding areas and generate secondary foci [17]. Although GBM cells present a pro-mesenchymal phenotype [18,19], different EMT (epithelial to mesenchymal transition)-like processes occur in GBM, which facilitate the acquisition of a more invasive phenotype. The most relevant is “glial to mesenchymal transition”, which correlates with the upregulation of mesenchymal markers and downregulation of astrocytic/glial markers such as GFAP, Olig2, FOXO1, or PDGFR [20,21,22]. GBM aggressiveness correlates with its infiltrative and disseminative capacity, since the tumour easily mixes with normal tissue, being even considered as a ‘‘whole-brain disease’’ [16]. Invasion is mainly performed along nerve tracts and meninges, but also through cerebrospinal fluid and brain blood vessels [16,23]. Disseminative cells hide in brain tumour-distant areas, where they cannot be detected or removed by surgery, rather than in perivascular areas, as seen on brain metastatic disseminated tumour cells.

Understanding the molecular mechanisms and signalling pathways that drive dissemination and tumorigenesis in GBM may lead not only to find better biomarkers of tumour status, but also novel therapeutic targets for its treatment. 

### 1.2. C3G: Generalities and Its Role in the Nervous System

**Figure 1:**
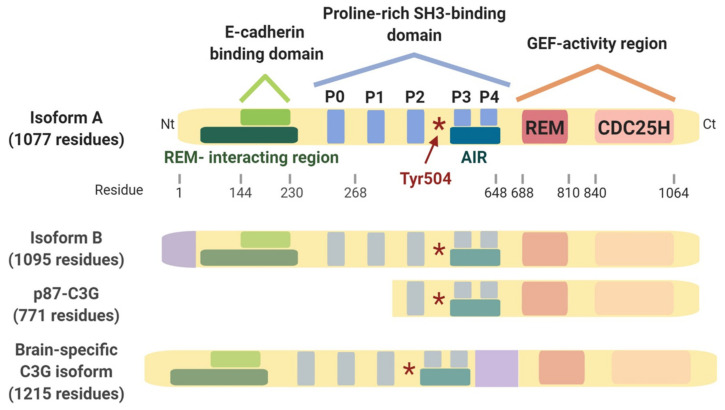
**C3G structure and isoforms.** C3G domains from N-terminal to C-terminal: E-cadherin-binding domain and REM-interacting region; SH3-binding proline-riche domains (P0–P4) and autoinhibitory region (AIR); REM and CDC25H, both responsible for C3G GEF activity. The asterisk indicates Tyr504, the most well-established residue susceptible of phosphorylation. In isoforms with more amino acids than isoform A, inserts are included in purple.

C3G (Crk SH3-domain-binding guanine-nucleotide-releasing factor), encoded by the *RAPGEF1* gene, is a GEF (guanine nucleotide exchange factor) for small GTPases, mainly Rap1. It also performs GEF-independent functions based on protein–protein interactions, involving its central polyproline (SH3-binding) and N-terminal regions (Figure 1). For example, C3G interacts with p130Cas, Cbl, and Abl to regulate leukemic cell adhesion [24]. However, all the mechanisms used by C3G to exert GEF-independent roles remain to be fully understood. C3G regulates proliferation, apoptosis/survival, actin remodelling, adhesion, migration, differentiation, and exocytosis [25,26]. C3G also plays an essential role during mouse embryogenesis [27], affecting, at least, the differentiation of skeletal muscle, the monocyte/macrophage and megakaryocyte/platelet lineages, the maturation of the vascular system, and different aspects of the nervous system development [25,28,29,30].

*RAPGEF1* is a ubiquitous, low-expressing gene that encodes a major isoform of 120 kDa, although multiple variants are expressed in a tissue-specific manner (Figure 1) [25]. C3G protein levels are higher in the brain compared to other tissues [31]. Embryonic brain is enriched in C3G isoforms containing non-catalytic regions [32] and a novel 175 kDa isoform, harbouring a 414 bp insert in the SH3-binding domain, and plays important roles in both embryonic and post-natal brain [33]. However, in adult brain, only the presence of the 120 kDa and 175 KDa isoforms has been confirmed [33], while it remains unknown whether other isoforms are also present.

Throughout mouse embryonic development, elevated levels of C3G are detected in the neural tube and cerebrocortical neuroepithelium [34]. In adult mouse brain, C3G is predominantly expressed in pyramidal neurons from the cerebral cortex, mitral cells of olfactory bulb, and hippocampal CA3 (Cornu Ammonis area 3) region [33]. It is known that C3G controls the size of the cerebral precursor population via Rap1-mediated inhibition of proliferative pathways such as β-catenin, Akt, and Ras-ERKs [34]. Moreover, C3G regulates neural precursor migration and differentiation. In particular, C3G participates in the control of precursor migration from the ventricular zone to form the cortical plate, in the multi-to-bipolar transition of neurons, in the formation of cortical axons and dendrites, and in the proper orientation of radial glial processes and their attachment to the pial surface [35,36]. Additionally, C3G is required for polarisation of hippocampal neurons [36] and contributes to sympathetic preganglionic neuron migration in the spinal cord [37].

Most of these actions of C3G are triggered by Reelin, an extracellular glycoprotein involved in cerebral cortex development [38]. It activates integrin α5β1 via ApoER2 (Apolipoprotein E Receptor 2) through an inside-out pathway involving Dab1-Crk/CrkL-C3G-Rap1. This pathway contributes to the proper neuronal positioning in the mature cortex [39,40]. Apart from the Reelin pathway, C3G participates in neural growth factor (NGF)-mediated sustained ERKs activation via Rap1, which is linked to differentiation of PC12 cells [41]. Interestingly, C3G is also involved in epidermal growth factor (EGF)-mediated transient ERKs activation in PC12 cells, inducing proliferation [41,42]. C3G also participates in pathways activated by different RTKs such as TrkA/B or Alk through diverse mediator proteins [43,44,45,46,47,48].

Despite the abundant literature on the role of C3G in the CNS, little is known about its involvement in nervous system pathologies including cancer.

## 2. Actions of C3G in GBM: What Is Different Compared to Other Tumours?

**Figure 2:**
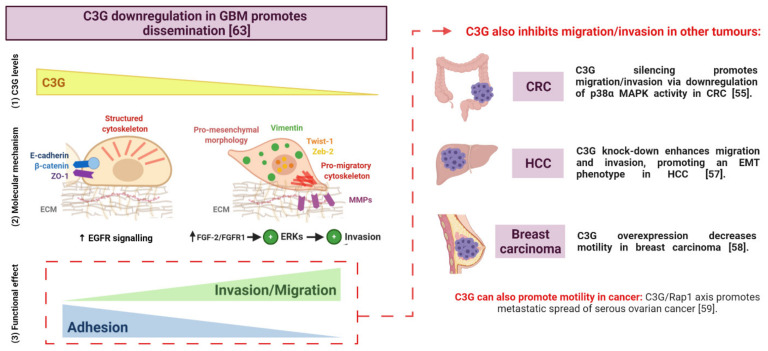
**Role of C3G in GBM cells dissemination as compared to other tumour types**. (1) C3G levels are high in healthy brain and they decrease along GBM progression [63]. (2) C3G knock-down enhances migration/invasion of GBM cells in a similar way that it does in other tumours [63]. Nevertheless, the mechanisms governing this pro-invasive effect are diverse. In GBM, ERKs are overactivated when C3G is downregulated, likely induced by the increased activation of FGF2-FGFR1 and other RTKs, which enhances invasion (left panel). (3) C3G is also a negative regulator of migration/invasion in other tumours, but not in all cancer types (right panel) [55,57,58]. In GBM, C3G is downregulated and this plays an important role enhancing tumour aggressiveness [63]. GBM: glioblastoma; CRC: colorectal carcinoma; HCC: hepatocellular carcinoma.

**Figure 3:**
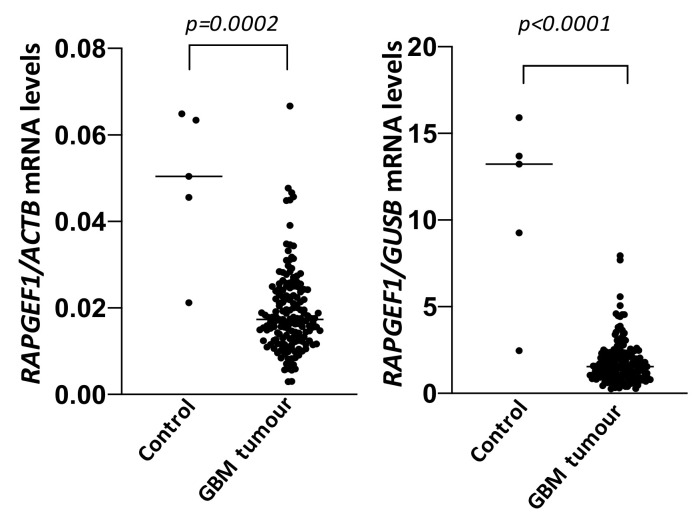
**RAPGEF1 mRNA levels in GBM patient tumour samples compared with healthy brain.** Analysis of RAPGEF1 expression in control (*n* = 5) and GBM patient samples (*n* = 166) using data from the TCGA database. RAPGEF1 mRNA levels normalised with ACTB (left panel) or GUSB (right panel). U-Mann–Whitney test was used to compare the data and p values are shown.

**Figure 4:**
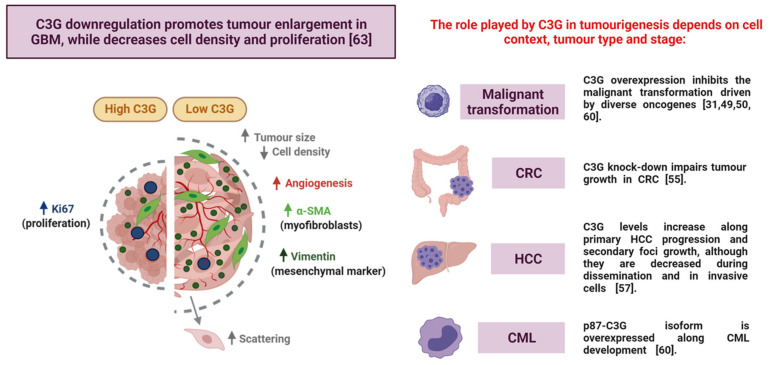
**Role played by C3G in GBM tumour growth compared to other tumourigenic processes.** In GBM, C3G knock-down increases the number of foci, cell scattering, tumour size, stroma infiltration, and angiogenesis, while decreases cell density, proliferation, and the number of tumour cells within the tumour (left panel). This differs from its actions in other tumours (right panel) [31,49,50,55,57,60]. GBM: glioblastoma; α-SMA: α-smooth muscle actin; CRC: colorectal carcinoma; HCC: hepatocellular carcinoma; CML: chronic myeloid leukaemia.

During the last few years, different studies have uncovered a function for C3G in cancer. However, it seems to play different roles depending on cell context, tumour type, and stage (Table 1 and Figure 2, Figure 3 and Figure 4). C3G inhibits malignant transformation of mouse fibroblasts induced by several oncogenes [31,49,50]. According to this function of C3G as a tumour suppressor, its expression is downregulated by promoter methylation in cervical-squamous cancer [51]. In contrast, the C3G-Rap pathway may contribute to the RET-induced transformed phenotype of thyroid cells [52]. In this line, C3G levels are upregulated in non-small cell lung cancer [53]. In addition, the *RAPGEF1* gene presents a somatic demethylation in colon, gastric, and ovarian cancer patients, which could be associated with an increased expression [54]. However, C3G plays a dual role in CRC (colorectal carcinoma) cells as demonstrated by in vitro and in vivo approaches [55]. C3G promotes tumour growth, while it inhibits invasion (Table 1). In HCC (hepatocellular carcinoma), the analyses of public databases indicate that *RAPGEF1* mRNA levels gradually increase in HCC patient samples during HCC progression up to stage III, which is associated with a reduced overall patient survival [56,57]. C3G protein levels are also increased in human HCC cell lines and mouse HCC models, promoting tumour growth through the activation of survival and proliferation, although it inhibits invasion [57] (Table 1). C3G overexpression also reduces the migration of breast carcinoma cells [58], while it seems to promote metastatic spread of serous ovarian cancer via Rap1 [59]. p87C3G, an isoform lacking the N-terminal region (Figure 1) is overexpressed in CML (chronic myeloid leukaemia) cell lines and patient samples and is associated with CML development [60]. Moreover, C3G downregulation enhances the STI-571 (imatinib mesylate) pro-apoptotic effect in CML cells [61]. In non-Hodgkin’s lymphoma patients, two missense mutations have been found in the C3G N-terminal region that causes Rap1 hyperactivation [62], which might lead to lymphoma progression (Table 1).

Based on the above comments, C3G is upregulated in a number of cancers, leading to tumour growth. In contrast, a reduction in C3G levels enhances migration and invasion, likely facilitating the dissemination of tumour cells (Figure 2).

According to public databases, and as previously described by Manzano et al. (2021), *RAPGEF1* mRNA levels are decreased in GBM patients compared to healthy brain tissue (Figure 3), although this is not associated with changes in overall survival [63], as occurs in other tumours. This could be due to the very low survival rate of GBM patients upon diagnosis. In agreement with the reduced *RAPGEF1* mRNA levels in GBM patient samples, protein levels of the main C3G isoform are also downregulated in several GBM cell lines compared to non-tumourigenic human astrocytes [63]. The 175 kDa novel brain-specific C3G isoform [33] was not detected as it is mainly expressed in postnatal and adult neurons, but not in astrocytes [33].

Even though C3G levels are reduced in GBM, a stronger downregulation of C3G in GBM cells by silencing using shRNAs promotes the acquisition of an enhanced mesenchymal phenotype. Hence, Vimentin levels are increased in *C3G* silenced GBM cells as well as their migratory and invasive capacity [63] (Figure 2). In this regard, it is important to mention that Vimentin upregulation is a well-established mesenchymal marker associated with poor prognosis in GBM [64,65,66,67]. Therefore, the reduction in C3G levels might represent a new biomarker of poor prognosis in GBM.

**Table 1:** Common and specific actions of C3G in different tumours and their mechanisms.

**Common** **C3G Effects and Mechanisms**	**Tumour**	**C3G Level** **mutations**	**Effect**	Mechanism	**Reference**
	Breast, CRC, HCC, and GBM	High/Medium	Decreases motility	Regulation of actin cytoskeleton	[55,57,58,63]
	CRC, HCC, and GBM	High/Medium	Inhibits migration and invasion	Blocking EMT like processes	[55,57,63]
	CRC, HCC, GBM, and CML	High/Medium	Promotes cell adhesion	Regulation of actin cytoskeleton	[55,57,60,63]
	CRC and HCC	Upregulated	Tumour growth promotion	Increase of cell proliferation and survival	[55,56,57]
**Specific** **C3G Effects and Mechanisms**	GBM	Downregulated	Migration and invasion enhancement	Activation of ERKs and FGFR1	[68,69]
	GBM	Downregulated	EGF/EGFR activation signalling impairment	Reduction of cell surface EGFR levels	[63]
	HCC	Upregulated	MET and HGF/MET signalling full activation	Contribution to the formation of MET signalling complexes	[57]
	CRC	High levels	Migration and invasion inhibition	Downregulation of p38α MAPK activity	[55]
	Serum ovarian cancer cells	High levels	Metastatic spread promotion	Via Rap1	[59]
	CML	p87C3G isoform upregulation	Disease development	Contribution to Bcr-Abl aberrant signalling	[60]
	Non-Hodgkin’s lymphoma	Y554H and M555K missense mutations in C3G AIR region	Rap1 hyperactivation	C3G auto-inhibitory mechanism disruption	[62]

CRC (colorectal cancer); HCC (hepatocellular carcinoma); GBM (glioblastoma); CML (chronic myeloid leukaemia); EMT (epithelial to mesenchymal transition).

This pro-migratory effect of *C3G* silencing is in agreement with the enhanced migration observed in *C3G*-silenced CRC cells [55] and HCC cells [57] and with the inhibitory effect of C3G overexpression on breast carcinoma cell migration [58] (Figure 2 and Table 1). Moreover, similarly to GBM cells, HCC cells showed a more pronounced mesenchymal phenotype upon C3G downregulation [57] and C3G-silenced CRC cells present MMP-2/9 activity upregulation, E-cadherin decrease, and ZO-1 internalisation [55]. However, it is still unclear whether reduced levels of C3G are enough for tumour cells to spread into their surroundings or to disseminate to distant tissues generating metastases in vivo. For instance, in HCC, C3G downregulation promotes cell dissemination and the generation of lung metastases, but the growth of these secondary tumours was associated to an upregulation of C3G [57].

The effect of C3G in GBM tumour growth is more complex than in other tumours. *C3G* silencing in GBM cells leads to the generation of larger tumours in xenograft assays and upon injection into the chick chorioallantoic membrane (CAM). However, proliferation is reduced and the number of tumour cells within the tumour is decreased [63] (Figure 4). This is also supported by in vitro anchorage-dependent and independent growth assays, but differs from the reduction in tumour size of CRC or HCC tumours generated by cells with C3G downregulation [55,57] (Figure 4). The enhanced migration of *C3G*-silenced GBM cells that favours cell scattering and the increased infiltration of endothelial cells and activated fibroblasts into the tumours would contribute to GBM tumour enlargement [63]. Novel future studies based on the generation of orthotopic xenografts will allow for further characterisation of the C3G contribution to GBM tumourigenesis.

Another interesting issue is the specific regulation of signalling pathways by C3G in GBM cells. The upregulation of ERK activity induced by *C3G* silencing is responsible for its main functional effects [63] (Figure 2), enhancing migration and invasion of GBM cells, as previously explained. This differs from the key role played by p38α MAPK in C3G knock-down CRC cells, mediating the enhanced migration and invasion [55]. The increased activation of several RTKs, and, in particular, FGFR1, a receptor associated with invasiveness in GBM [68,69], seems to play an important role in promoting invasion through ERK activation [63]. In contrast, the activity of EGFR and other RTKs is impaired when *C3G* is downregulated in GBM cells. Hence, *C3G* knock-down decreases EGFR signalling and functionality by reducing cell surface EGFR levels through inhibition of its recycling [63]. This represents a new mechanism used for C3G to regulate EGFR signalling in GBM cells, which might be a more general mechanism involved in the control of the trafficking of other proteins. In any case, this defective EGFR signalling might contribute to the resistance to anti-EGFR therapy in patients with low levels of C3G. In HCC cells, C3G is also required for the full activation of MET, but through the regulation of signalling complex formation [57].

The role of C3G controlling RTK activation in GBM cells is a novel mechanism that should be further studied. It is known that the C3G-Rap1 axis promotes survival and differentiation of human IMR-32 neuroblastoma cells in response to NGF, while inhibiting their proliferation [45,47]. Moreover, oncogenic forms of the ALK receptor tyrosine kinase engage the CrkL-C3G-Rap1 pathway to induce proliferation of SK-N-SH and SH-SY5Y neuroblastoma cell lines, although ALK also induces neurite outgrowth of PC12 cells [46]. In addition, while normal brain tissue shows cytoplasmic expression of C3G, IMR-32 neuroblastoma cells show significantly higher levels of nuclear C3G [33]. Considering these results and those recently published by Manzano et al. (2021), C3G may act as a wide-range regulator of RTK activation and functionality in tumours from the nervous system, affecting tumour proliferation, expression, and dissemination. Nevertheless, more studies are needed to confirm this hypothesis.

Another important issue that remains undetermined is whether C3G acts through GEF dependent or independent mechanisms to regulate GBM and Rap1 activation status in these tumours.

## 3. Functions of the Main C3G Target, Rap, in GBM

The Rap (Ras-related protein) subfamily belongs to the Ras superfamily of small G proteins and includes two subtypes, Rap1 and Rap2 (with a 60% homology), and five isoforms: Rap1A, Rap1B, Rap2A, Rap2B, and Rap2C [70]. As a small GTPase, Rap activation is induced by a conformational change that allows for GTP binding, facilitating GDP release. This is catalysed by GEFs such as C3G, while GAPs increase endogenous Rap GTPase activity, leading to the hydrolysis of bound GTP to GDP. Thus, specific GEFs and GAPs modulate Rap activation, signal duration, and localisation in response to different stimuli.

The function of Rap proteins as tumour promoters or suppressors is controversial, with different studies describing paradoxical results. This may be dependent on cell type and/or activation insights and different Rap isoforms may lead to opposite outcomes. Depending on the cellular context, Rap1 can regulate cell–cell and/or cell–ECM (extracellular matrix) adhesion. These functions of Rap1 depend on its ability to achieve an intracellular control of integrin signalling [71].

High-grade astrocytomas frequently overexpress Rap1 [72] and the expression of Rap1B was reported to be increased with pathological progression [73]. Moreover, GBM tumours show higher Rap1 activity than lower grade astrocytomas or healthy brain [72]. In addition, Rap1A expression is elevated in GBM and correlates with higher tumour grade [74]. Moreover, higher levels of Rap2B are associated with a poorer survival of patients with low grade gliomas [75]. Despite all of this, the role of Rap proteins in GBM is still unclear, with different members of the family associated with diverse, even opposite, functions.

On one hand, Rap1 has been shown to promote proliferation in GBM cells [76,77]. In response to thrombin, RhoA activates Rap1 and promotes integrin signalling and cell proliferation [77]. Dopamine Receptor D2 (DRD2) induces GBM proliferation through a GNAI2/Rap1/Ras/ERK signalling axis [76]. Moreover, Rap1 regulates GSC tumour initiating capacity and stemness by controlling their anchorage to the perivascular niche of malignant glioma through activation of integrin signalling [78]. In agreement with this, a recent report has shown that Rap2B also induces glioma cell proliferation [75]. On the other hand, the Epac1/Rap1 axis, in combination with PKA, has been shown to promote GBM cell death in response to Rolipram, which upregulates intracellular cAMP levels [79]. Nevertheless, miR-128, miR-149, and miR-181 increase GBM chemosensitivity to TMZ by inhibiting Rap1B-mediated cytoskeletal remodelling [80,81]. Hence, Rap protein’s role in GBM proliferation and survival seems to depend on the stimuli and the isoform involved.

Rap1 has also been shown to mediate glioma invasion and migration in response to PDGF-BB downstream of kinase 1 (DOK1) and p130Cas activation [82]. Overexpression of RasGRP3, a GEF common for Ras and Rap subfamilies, also promotes migration and invasion of glioma cells [83]. Furthermore, Rap1A has recently been shown to promote GSCs migration [74]. Rap2B has also been described to induce metalloprotease activation, promoting glioma cell invasion and migration through regulation of the ERKs’ pathway [75]. In agreement with this, Rap1B was identified in a competitive endogenous RNA (ceRNA) network as one of the six genes that act as a marker of GBM mesenchymal subtype and positively regulate invasiveness [84]. Previous reports have also shown that Rap1B regulates the GBM cytoskeleton [80,81]. In contrast, Rap1B can also cooperate with PTEN to negatively regulate GBM invasion through inhibition of Rac in response to isoproterenol [85]. Hence, the role of Rap1B in GBM cell invasion and migration might depend on cell context and/or GBM subtype. Rap2A can also act as an inhibitor of glioma cell invasion and migration in response to deregulated microRNA editing [86]. In a recent report, BM-MSCs (Bone Marrow-Derived Mesenchymal Stem Cells)-conditioned media was shown to promote glioma cell migration and invasion. Rap2C was identified as one of the proteins downregulated in glioma cells treated with BM-MSC-conditioned media, suggesting it might negatively regulate glioma cell invasion [87].

In summary, the roles played by the different Rap subfamily members in GBM are not clear, so further studies should be performed to clarify them. Epac2, one of the most predominant RapGEFs in the brain, has recently been proposed as a negative regulator of glioma invasion and MMP-2 activity [88], similarly to what we observed for C3G [63]. This suggests that Rap regulators might play a relevant role in GBM, and that C3G and Epac2 RapGEFs could collaborate to inhibit GBM dissemination acting through Rap, although C3G GEF independent functions could also be relevant in GBM.

## 4. C3G in Clinics: Present and Future Perspectives

Throughout this review, it has been shown that C3G regulates diverse functions all along the life span from embryonic development until adulthood, acting through GEF-dependent and -independent mechanisms. As described in the introduction, C3G plays specific key roles in tissue differentiation and remodelling [25,28,29,30] as well as in cell proliferation, apoptosis/survival, or cytoskeleton organisation involved in adhesion and migration processes [25,26].

It is important to highlight the relevance of a fine tune regulation of C3G levels across tissues [25] and how their dysregulation is associated with different tumour types, as observed in multiple studies: non-small lung carcinoma [53], CML [60], neuroblastoma and pheochromocytoma [46], breast carcinoma [58], ovarian cancer [59], CRC [55], HCC [56,57], non-Hodgkins lymphoma [62], and GBM [63].

Despite its probed role in some of the most aggressive carcinomas, up to now, C3G has not been included as a target for cancer therapy in any clinical trial. This can be explained by different factors: (i) C3G seems to play different roles depending on cell context, tumour type, and stage, so C3G should be specifically over- or downregulated in a unique way depending on the affected organ; and (ii) C3G is a ubiquitous low-expressed protein [25], suggesting a fine tune regulation of C3G expression that makes it very difficult to develop efficient, safe, and highly controlled therapies for targeting or regulating C3G levels. In addition, due to the existence of different isoforms, some of them are still not well characterised, it is even more complex to use C3G as a good target for therapy. However, C3G could represent a new biomarker for GBM diagnosis and prognosis. In addition, the quantification of C3G levels might be a useful tool for a personalised treatment of GBM patients. It would be expected that GBM patients with lower levels of C3G do not respond to anti-EGFR therapy. In contrast, both FGFR1, and ERKs might represent alternative therapeutic targets for GBM patients with low levels of C3G based on the upregulation of their activities. Future studies aimed to further explore C3G function in RTK regulation will be of great interest for the design of novel and personalised therapeutic strategies. It will also be important to determine how C3G levels affect the response to TMZ or other chemotherapeutic agents. In addition, the characterisation of C3G actions on the stemness capacity of GBM cells and the underlying mechanisms will also contribute to the identification of new targets.

Reviewing the literature and the African, Australian New Zealand, European, and North American Clinical Trials Registries, we found a few trials that attempted to alter molecules involved in C3G signalling pathways. However, none of them were focused on GBM. Two trials analysed the Src pathway quantifying Crk-L, a protein that interacts with C3G protein: (i) after a combinatorial therapy consisting on the tyrosine kinase inhibitor (TKI) dasatinib, paclitaxel (microtubule inhibitor), and carboplatin (alkylating antineoplastic agent) in patients with advanced or recurrent ovarian, peritoneal, or tubal carcinoma (NCT00672295), and (ii) after the treatment of Philadelphia chromosome positive CML patients with bosutinib, a Src tyrosine kinase inhibitor (NCT00261846).

The former assay has not provided information since “the SRC signature” could not be applied directly as a predictive model in this dataset because of the limited sample size [89]. However, the later study has shown promising data about Crk-L phosphorylation (i.e., inhibition of tumour cell growth when the medium dose (500 mg bosutinib) was administered) (Table 2).

A couple of trials have been developed with Rap1 as a therapeutic target: (i) the humanised monoclonal antibody against the interleukin-6 receptor (IL6R) tocilizumab, which attempted to modulate Rap1 expression and, therefore, reduce the acute graft-versus-host disease progression or cancer relapse after allogeneic hematopoietic stem cell transplantation (NCT02206035), even though the trial has been completed, the results have not been shared or published yet; (ii) in individuals undergoing autologous hematopoietic cell transplant to treat multiple myeloma, the nonspecific beta-blocker propranolol, which impairs adenylate cyclase activation and, hence, cAMP generation, was administered to analyse whether membrane localisation and prenylation of Rap1 in PBMCs (peripheral blood mononuclear cell) could be altered, and could therefore show a therapeutic response (NCT02535182) (Table 2). Up to today, it is unclear what the effect of propranolol is on Rap1.

So, is there a future possibility for C3G to be included in a clinical trial? We think that with the exception of a highly controlled/regulated tissue specific gene therapy/edition approach, C3G could be used as a biomarker for GBM diagnosis and prognosis. The quantification of C3G levels might be a useful tool for a personalised treatment of GBM patients. Then, the question immediately arises, how to detect and quantify this protein in a cancer like GBM? The liquid-biopsy technique is the pole-sitter. In GBM, liquid-biopsy offers some clear advantages in comparison with tumour tissue extraction or MR imaging techniques and biopsy, since it is less invasive, allowing a more frequent follow-up, and can offer a whole picture rather than a tumour localised region. GBM, as other solid tumours, drop diverse contents to the cellular and humoral microenvironment that can be widely distributed via the vascular system or CSF (cerebrospinal fluid) [90]. Therefore, tumour markers could be detected in these fluids as well as in urine or saliva samples [91,92]. In GBM, liquid-biopsy has been able to detect CTCs (circulating tumour cells), extracellular vesicles, cell-free nucleic acids, metabolites, proteins, and TEPs (tumour-educated platelets) [91] and some of these samples could potentially contain C3G. Manzano et al. (2021) [63] showed that *C3G* expression decreased along with the evolution of GBM malignancy. Moreover, *C3G*-silenced GBM cells demonstrated a more invasive and less adhesive phenotype. Thus, GBM-derived CTCs should present lower levels of C3G and its differential expression levels along the time would have a prognosis value for GBM tumours. In contrast, higher levels of C3G in mouse TEPs are associated with the promotion of angiogenic processes in tumours by inhibiting the secretion of the antiangiogenic factor thrombospondin-1 (TSP-1) [26]. This would correlate with tumour progression and, hence, prognosis. It would be interesting to test whether this also operates in GBM and if platelet C3G represents a new potential biomarker and/or therapeutic target in GBM.

## 5. Concluding Remarks

As explained above, a downregulation of *RAPGEF1* mRNA levels in human GBM tumours and a decrease in C3G protein levels in GBM cells have recently been discovered [63]. Moreover, C3G silencing enhances invasiveness and a pro-mesenchymal phenotype. This role of C3G as a negative regulator of invasiveness of GBM cells is a common effect of C3G in several cancer types [55,57,58] and in cells from the nervous system [34,35].

However, the function of C3G in GBM tumourigenesis is more complex. C3G downregulation leads to the generation of bigger tumours, even though proliferation is reduced [63]. This differs from other tumours such as CRC or HCC, in which C3G downregulation reduces tumour size [55,57] and might be caused by GBM cell scattering and the infiltration of cells from the stroma.

It remains undetermined whether C3G GEF activity could be involved in its actions on GBM and whether Rap proteins are C3G mediators. As widely reviewed above, published studies have revealed different and even opposite functions for Rap proteins in GBM [75,76,77,79,82,84], showing its dependency on cell context. Nevertheless, Epac2 and C3G, two important RapGEFs in the brain, seem to play similar roles in GBM [63,88]. This establishes a connection between these GEFs and their target GTPases and highlights the importance of the regulation of the activity of Rap proteins and their regulators in these tumours.

All the above referred effects of C3G downregulation in GBM indicate that C3G levels could be used in clinic. Nowadays, the use of C3G as a target for GBM treatment remains discouraged. However, a novel prognostic GBM signature could be generated to evaluate its progression and to select effective therapies, which would include the quantification of C3G and Vimentin levels, ERK activation, and/or the evaluation of the switching from EGFR to FGFR signalling, among others.

Compilation of the interventional clinical trials registered at the Pan African Clinical Trials Registry. Available online: https://pactr.samrc.ac.za/Default.aspx (accessed on 8 July 2021), Australian New Zealand Clinical Trials Reg-istry. Available online: http://www.anzctr.org.au/Default.aspx (accessed on 8 July 2021), EU Clinical Trials Register. Available online: https://www.clinicaltrialsregister.eu (accessed on 8 July 2021), and the U.S. National Library of Medicine. Available online: https://clinicaltrials.gov (accessed on 8 July 2021). Only interventional trials in which a therapeutic drug was tested are listed.

## Figures and Tables

**Table 2:** Compilation of the interventional clinical trials registered studying molecules involved in C3G signalling pathways.

Clinical Trial ID	Phase	Title	Disease	Intervention	Number of Patients	Trial design	Outcome	Start Date	Status
NCT00261846	1/2	Study Evaluating SKI-606 (Bosutinib) In Philadelphia Chromosome Positive Leukaemias	Myeloid Leukaemia	Bosutinib	571	Single-arm, open-label	Number of Participants with Dose Limiting Toxicity (DLT) (Time Frame: Part 1 Baseline up to Day 28) and pharmacokinetics	January 2006	Completed
NCT00672295	1	PH I SRC Kinase, Dasatinib Combo Paclitaxel & Carboplatin in Pts w Ovarian, Peritoneal, & Tubal Cancer	Ovarian Cancer; Peritoneal Cancer; Fallopian Tube Cancer	Dasatinib, Paclitaxel, and Carboplatin	11	Single-arm, open-label	To determine maximal tolerated dose (MTD) of dasatinib in combination with paclitaxel and carboplatin during the first cycle of treatment (Time Frame: 6 months)	August 2007	Completed
NCT02206035	2	Phase II Open-Label Trial of Tacrolimus/Methotrexate and Tocilizumab for the Prevention of Acute Graft-Versus-Host Disease After Allogeneic Hematopoietic Stem Cell Transplantation	Hematopoietic Stem Cell Transplantation	Tacrolimus, Methotrexate, Tocilizumab	35	Single-arm, open-label	Grade II-IV aGVHD-free survival (Time Frame: Day 180). Comparison of grade II-IV a GVHD-free survival at day 180 between recipients of Tac/MTX/Toc to contemporary CIBMTR controls.	December 2014	Completed
NCT02535182	2	Pilot Study Using Propranolol to Promote Prenylation of GTPase Rap1b in Hematopoietic Stem Cell Transplant Recipients	Hematopoietic Stem Cell Transplantation	Propranolol	25	Two-arm, randomised, double-blind, placebo-controlled	Prenylation levels in response to propranolol in a population of patients undergoing autologous hematopoietic stem cell transplantation (Time Frame: 1 year)	August 2015	Completed

## Data Availability

Not applicable.

## References

[1] Louis D.N., Ohgaki H., Wiestler O.D., Cavenee W.K., Burger P.C., Jouvet A., Scheithauer B.W., Kleihues P. (2007). The 2007 WHO Classification of Tumours of the Central Nervous System. Acta Neuropathol..

[2] Ostrom Q.T., Bauchet L., Davis F.G., Deltour I., Fisher J.L., Eastman Langer C., Pekmezci M., Schwartzbaum J.A., Turner M.C., Walsh K.M. (2014). The epidemiology of glioma in adults: A “state of the science” review. Neuro-Oncology.

[3] Tian M., Ma W., Chen Y., Yu Y., Zhu D., Shi J., Zhang Y. (2018). Impact of gender on the survival of patients with glioblastoma. Biosci. Rep..

[4] Stupp R., Mason W.P., van den Bent M.J., Weller M., Fisher B., Taphoorn M.J.B., Belanger K., Brandes A.A., Marosi C., Bogdahn U. (2005). Radiotherapy plus Concomitant and Adjuvant Temozolomide for Glioblastoma. N. Engl. J. Med..

[5] Louis D.N., Perry A., Reifenberger G., von Deimling A., Figarella-Branger D., Cavenee W.K., Ohgaki H., Wiestler O.D., Kleihues P., Ellison D.W. (2016). The 2016 World Health Organization Classification of Tumors of the Central Nervous System: A summary. Acta Neuropathol..

[6] Louis D.N., Perry A., Wesseling P., Brat D.J., Cree I., Figarella-Branger D., Hawkins C., Ng H.K., Pfister S.M., Reifenberger G. (2021). The 2021 WHO Classification of Tumors of the Central Nervous System: A summary. Neuro-Oncology.

[7] Phillips H.S., Kharbanda S., Chen R., Forrest W.F., Soriano R.H., Wu T.D., Misra A., Nigro J.M., Colman H., Soroceanu L. (2006). Molecular subclasses of high-grade glioma predict prognosis, delineate a pattern of disease progression, and resemble stages in neurogenesis. Cancer Cell.

[8] Verhaak R.G.W., Hoadley K.A., Purdom E., Wang V., Qi Y., Wilkerson M.D., Miller C.R., Ding L., Golub T., Mesirov J.P. (2010). Integrated Genomic Analysis Identifies Clinically Relevant Subtypes of Glioblastoma Characterized by Abnormalities in PDGFRA, IDH1, EGFR, and NF1. Cancer Cell.

[9] Behnan J., Finocchiaro G., Hanna G. (2019). The landscape of the mesenchymal signature in brain tumours. Brain.

[10] Neftel C., Laffy J., Filbin M.G., Hara T., Shore M.E., Rahme G.J., Richman A.R., Silverbush D., Shaw M.L., Hebert C.M. (2019). An Integrative Model of Cellular States, Plasticity, and Genetics for Glioblastoma. Cell.

[11] Hara T., Chanoch-Myers R., Mathewson N.D., Myskiw C., Atta L., Bussema L., Eichhorn S.W., Greenwald A.C., Kinker G.S., Rodman C. (2021). Interactions between cancer cells and immune cells drive transitions to mesenchymal-like states in glioblastoma. Cancer Cell.

[12] TCGA (2008). Comprehensive genomic characterization defines human glioblastoma genes and core pathways. Genomics.

[13] Pearson J., Regad T. (2017). Targeting cellular pathways in glioblastoma multiforme. Signal Transduct. Target. Ther..

[14] Lathia J.D., Mack S.C., Mulkearns-Hubert E.E., Valentim C.L., Rich J.N. (2015). Cancer stem cells in glioblastoma. Genes Dev..

[15] Liebelt B.D., Shingu T., Zhou X., Ren J., Shin S., Hu J. (2016). Glioma Stem Cells: Signaling, Microenvironment, and Therapy. Stem Cells Int..

[16] Agarwal S., Sane R., Oberoi O., Ohlfest J.R., Elmquist W.F. (2011). Delivery of Molecularly Targeted Therapy to Malignant Glioma, a Disease of the Whole Brain. Expert Rev. Mol. Med..

[17] Lun M., Lok E., Gautam S., Wu E., Wong E.T. (2011). The natural history of extracranial metastasis from glioblastoma multiforme. J. Neuro-Oncol..

[18] Tso C.-L., Shintaku P., Chen J., Liu Q., Liu J., Chen Z., Yoshimoto K., Mischel P.S., Cloughesy T.F., Liau L.M. (2006). Primary Glioblastomas Express Mesenchymal Stem-Like Properties. Mol. Cancer Res..

[19] Iser I.C., Pereira M.B., Lenz G., Wink M.R. (2017). The Epithelial-to-Mesenchymal Transition-Like Process in Glioblastoma: An Updated Systematic Review and In Silico Investigation. Med. Res. Rev..

[20] Mahabir R., Tanino M., Elmansuri A., Wang L., Kimura T., Itoh T., Ohba Y., Nishihara H., Shirato H., Tsuda M. (2014). Sustained elevation of Snail promotes glial-mesenchymal transition after irradiation in malignant glioma. Neuro-Oncology.

[21] Matias D., Balça-Silva J., Dubois L.G.F., Pontes B., Ferrer V.P., Rosário L., Carmo A., Echevarria-Lima J., .Sarmento-Ribeiro A.B., Lopes M.C. (2017). Dual treatment with shikonin and temozolomide reduces glioblastoma tumor growth, migration and glial-to-mesenchymal transition. Cell. Oncol..

[22] Chen C., Han G., Li Y., Yue Z., Wang L., Liu J. (2019). FOXO1 associated with sensitivity to chemotherapy drugs and glial-mesenchymal transition in glioma. J. Cell. Biochem..

[23] Van Meir E.G., Hadjipanayis C.G., Norden A.D., Shu H.-K., Wen P.Y., Olson J.J. (2010). Exciting New Advances in Neuro-Oncology: The Avenue to a Cure for Malignant Glioma. CA A Cancer J. Clin..

[24] Maia V., Ortiz-Rivero S., Sanz M., Gutierrez-Berzal J., Álvarez-Fernández I., Gutiérrez-Herrero S., De Pereda J.M., Porras A., Guerrero C. (2013). C3G forms complexes with Bcr-Abl and p38α MAPK at the focal adhesions in chronic myeloid leukemia cells: Implication in the regulation of leukemic cell adhesion. Cell Commun. Signal..

[25] Radha V., Mitra A., Dayma K., Sasikumar K. (2011). Signalling to actin: Role of C3G, a multitasking guanine-nucleotide-exchange factor. Biosci. Rep..

[26] Martín-Granado V., Ortiz-Rivero S., Carmona R., Gutiérrez-Herrero S., Barrera M., San-Segundo L., Sequera C., Perdiguero P., Lozano F., Martín-Herrero F. (2017). C3G promotes a selective release of angiogenic factors from activated mouse platelets to regulate angiogenesis and tumor metastasis. Oncotarget.

[27] Ohba Y., Ikuta K., Ogura A., Matsuda J., Mochizuki N., Nagashima K., Kurokawa K., Mayer B.J., Maki K., Miyazaki J. (2001). Requirement for C3G-dependent Rap1 activation for cell adhesion and embryogenesis. EMBO J..

[28] Voss A.K., Gruss P., Thomas T. (2003). The guanine nucleotide exchange factor C3G is necessary for the formation of focal adhesions and vascular maturation. Development.

[29] Kumar K.S., Ramadhas A., Nayak S., Kaniyappan S., Dayma K., Radha V. (2015). C3G (RapGEF1), a regulator of actin dynamics promotes survival and myogenic differentiation of mouse mesenchymal cells. Biochim. Biophys. Acta Mol. Cell Res..

[30] Ortiz-Rivero S., Baquero C., Hernández-Cano L., Roldán-Etcheverry J.J., Gutiérrez-Herrero S., Fernández-Infante C., Martín-Granado V., Anguita E., De Pereda J.M., Porras A. (2018). C3G, through its GEF activity, induces megakaryocytic differentiation and proplatelet formation. Cell Commun. Signal..

[31] Guerrero C., Fernandez-Medarde A., Rojas J.M., Font de Mora J., Esteban L.M., Santos E. (1998). Transformation suppressor activity of C3G is independent of its CDC25-homology domain. Oncogene.

[32] Cheerathodi M., Vincent J.J., Ballif B.A. (2015). Quantitative comparison of CrkL-SH3 binding proteins from embryonic murine brain and liver: Implications for developmental signaling and the quantification of protein species variants in bottom-up proteomics. J. Proteom..

[33] Sriram D., Chintala R., Parthasaradhi B.V.V., Nayak S.C., Mariappan I., Radha V. (2020). Expression of a novel brain specific isoform of C3G is regulated during development. Sci. Rep..

[34] Voss A.K., Krebs D.L., Thomas T. (2006). C3G regulates the size of the cerebral cortex neural precursor population. EMBO J..

[35] Voss A.K., Britto J.M., Dixon M.P., Sheikh B., Collin C., Tan S.-S., Thomas T. (2008). C3G regulates cortical neuron migration, preplate splitting and radial glial cell attachment. Development.

[36] Shah B., Lutter D., Bochenek M.L., Kato K., Tsytsyura Y., Glyvuk N., Sakakibara A., Klingauf J., Adams R.H., Püschel A.W. (2016). C3G/Rapgef1 Is Required in Multipolar Neurons for the Transition to a Bipolar Morphology during Cortical Development. PLoS ONE.

[37] Yip Y.P., Thomas T., Voss A.K., Yip J.W. (2012). Migration of sympathetic preganglionic neurons in the spinal cord of a C3G-deficient mouse suggests that C3G acts in the reelin signaling pathway. J. Comp. Neurol..

[38] Bock H.H., May P. (2016). Canonical and Non-canonical Reelin Signaling. Front. Cell. Neurosci..

[39] Ballif B.A., Arnaud L., Arthur W.T., Guris D., Imamoto A., Cooper J.A. (2004). Activation of a Dab1/CrkL/C3G/Rap1 Pathway in Reelin-Stimulated Neurons. Curr. Biol..

[40] Sekine K., Kawauchi T., Kubo K.-I., Honda T., Herz J., Hattori M., Kinashi T., Nakajima K. (2012). Reelin Controls Neuronal Positioning by Promoting Cell-Matrix Adhesion via Inside-Out Activation of Integrin α5β1. Neuron.

[41] Kao S.-C., Jaiswal R.K., Kolch W., Landreth G.E. (2001). Identification of the Mechanisms Regulating the Differential Activation of the MAPK Cascade by Epidermal Growth Factor and Nerve Growth Factor in PC12 Cells. J. Biol. Chem..

[42] Lua L., Anneréna C., Reedquist K.A., Bos J.L., Welsh M. (2000). NGF-Dependent Neurite Outgrowth in PC12 Cells Overexpressing the Src Homology 2-Domain Protein Shb Requires Activation of the Rap1 Pathway. Exp. Cell Res..

[43] Wu C., Lai C.-F., Mobley W.C. (2001). Nerve Growth Factor Activates Persistent Rap1 Signaling in Endosomes. J. Neurosci..

[44] Arévalo J.C., Pereira D.B., Yano H., Teng K., Chao M.V. (2006). Identification of a Switch in Neurotrophin Signaling by Selective Tyrosine Phosphorylation. J. Biol. Chem..

[45] Radha V., Rajanna A., Gupta R.K., Dayma K., Raman T. (2008). The guanine nucleotide exchange factor, C3G regulates differentiation and survival of human neuroblastoma cells. J. Neurochem..

[46] Schönherr C., Yang H.-L., Vigny M., Palmer R.H., Hallberg B. (2010). Anaplastic lymphoma kinase activates the small GTPase Rap1 via the Rap1-specific GEF C3G in both neuroblastoma and PC12 cells. Oncogene.

[47] Mitra A., Kalayarasan S., Gupta V., Radha V. (2011). TC-PTP Dephosphorylates the Guanine Nucleotide Exchange Factor C3G (RapGEF1) and Negatively Regulates Differentiation of Human Neuroblastoma Cells. PLoS ONE.

[48] Dar M.I., Jan S., Reddy G.L., Wani R., Syed M., Mohd J.D., Sawant S.D., Vishwakarma R.A., Syed S.H. (2020). Differentiation of human neuroblastoma cell line IMR-32 by sildenafil and its newly discovered analogue IS00384. Cell. Signal..

[49] Guerrero C., Martín-Encabo S., Fernández-Medarde A., Santos E. (2004). C3G-mediated suppression of oncogene-induced focus formation in fibroblasts involves inhibition of ERK activation, cyclin A expression and alterations of anchorage-independent growth. Oncogene.

[50] Martín-Encabo S., Santos E., Guerrero C. (2007). C3G mediated suppression of malignant transformation involves activation of PP2A phosphatases at the subcortical actin cytoskeleton. Exp. Cell Res..

[51] Okino K., Nagai H., Nakayama H., Doi D., Yoneyama K., Konishi H., Takeshita T. (2006). Inactivation of Crk SH3 domain-binding guanine nucleotide-releasing factor (C3G) in cervical squamous cell carcinoma. Int. J. Gynecol. Cancer.

[52] De Falco V., Castellone M., De Vita G., Cirafici A.M., Hershman J.M., Guerrero C., Fusco A., Melillo R.M., Santoro M. (2007). RET/Papillary Thyroid Carcinoma Oncogenic Signaling through the Rap1 Small GTPase. Cancer Res..

[53] Hirata T., Nagai H., Koizumi K., Okino K., Harada A., Onda M., Nagahata T., Mikami I., Hirai K., Haraguchi S. (2004). Amplification, up-regulation and over-expression of C3G (CRK SH3 domain-binding guanine nucleotide-releasing factor) in non-small cell lung cancers. J. Hum. Genet..

[54] Perucho M., Samuelsson J., Alonso S., Ruiz-Larroya T., Cheung T.H., Wong Y.F. (2011). Frequent somatic demethylation of RAPGEF1/C3G intronic sequences in gastrointestinal and gynecological cancer. Int. J. Oncol..

[55] Priego N., Arechederra M., Sequera C., Bragado P., Vázquez-Carballo A., Gutierrez-Uzquiza A., Martín-Granado V., Ventura J.-J., Kazanietz M.G., Guerrero C. (2016). C3G knock-down enhances migration and invasion by increasing Rap1-mediated p38α activation, while it impairs tumor growth through p38α-independent mechanisms. Oncotarget.

[56] Sequera C., Manzano S., Guerrero C., Porras A. (2018). How Rap and its GEFs control liver physiology and cancer development. C3G alterations in human hepatocarcinoma. Hepatic Oncol..

[57] Sequera C., Bragado P., Manzano S., Arechederra M., Richelme S., Gutiérrez-Uzquiza A., Sánchez A., Maina F., Guerrero C., Porras A. (2020). C3G Is Upregulated in Hepatocarcinoma, Contributing to Tumor Growth and Progression and to HGF/MET Pathway Activation. Cancers.

[58] Dayma K., Radha V. (2011). Cytoskeletal remodeling by C3G to induce neurite-like extensions and inhibit motility in highly invasive breast carcinoma cells. Biochim. Biophys. Acta Mol. Cell Res..

[59] Che Y.-L., Luo S.-J., Li G., Cheng M., Gao Y.-M., Li X.-M., Dai J.-M., He H., Wang J., Peng H.-J. (2015). The C3G/Rap1 pathway promotes secretion of MMP-2 and MMP-9 and is involved in serous ovarian cancer metastasis. Cancer Lett..

[60] Gutiérrez-Berzal J., Castellano E., Martín-Encabo S., Gutiérrez-Cianca N., Hernández J.M., Santos E., Guerrero C. (2006). Characterization of p87C3G, a novel, truncated C3G isoform that is overexpressed in chronic myeloid leukemia and interacts with Bcr-Abl. Exp. Cell Res..

[61] Maia V., Sanz M., Gutierrez-Berzal J., De Luis A., Gutierrez-Uzquiza A., Porras A., Guerrero C. (2009). C3G silencing enhances STI-571-induced apoptosis in CML cells through p38 MAPK activation, but it antagonizes STI-571 inhibitory effect on survival. Cell. Signal..

[62] Carabias A., Gómez-Hernández M., De Cima S., Rodríguez-Blázquez A., Morán-Vaquero A., González-Sáenz P., Guerrero C., De Pereda J.M. (2020). Mechanisms of autoregulation of C3G, activator of the GTPase Rap1, and its catalytic deregulation in lymphomas. Sci. Signal..

[63] Manzano S., Gutierrez-Uzquiza A., Bragado P., Sequera C., Herranz Ó., Rodrigo-Faus M., Jauregui P., Morgner S., Rubio I., Guerrero C. (2021). C3G downregulation induces the acquisition of a mesenchymal phenotype that enhances aggressiveness of glioblastoma cells. Cell Death Dis..

[64] Ivaska J. (2011). Vimentin: Central hub in EMT induction?. Small GTPases.

[65] Lin L., Wang G., Ming J., Meng X., Han B., Sun B., Cai J., Jiang C. (2016). Analysis of expression and prognostic significance of vimentin and the response to temozolomide in glioma patients. Tumor Biol..

[66] Zhao J., Zhang L., Dong X., Liu L., Huo L., Chen H. (2018). High Expression of Vimentin is Associated With Progression and a Poor Outcome in Glioblastoma. Appl. Immunohistochem. Mol. Morphol..

[67] Nowicki M.O., Hayes J.L., Chiocca E.A., Lawler S.E. (2019). Proteomic Analysis Implicates Vimentin in Glioblastoma Cell Migration. Cancers.

[68] Loilome W., Joshi A.D., Ap Rhys C.M.J., Piccirillo S.G.M., Angelo V.L., Gallia G.L., Riggins G.J. (2009). Glioblastoma cell growth is suppressed by disruption of fibroblast growth factor pathway signaling. J. Neuro-Oncol..

[69] Jimenez-Pascual A., Siebzehnrubl F.A. (2019). Fibroblast Growth Factor Receptor Functions in Glioblastoma. Cells.

[70] Guo X.-X., An S., Yang Y., Liu Y., Hao Q., Xu T.-R. (2016). Rap-Interacting Proteins are Key Players in the Rap Symphony Orchestra. Cell. Physiol. Biochem..

[71] Boettner B., Van Aelst L. (2009). Control of cell adhesion dynamics by Rap1 signaling. Curr. Opin. Cell Biol..

[72] Lau N., Uhlmann E.J., Von Lintig F.C., Nagy A., Boss G.R., Gutmann D.H., Guha A. (2003). Rap1 activity is elevated in malignant astrocytomas independent of tuberous sclerosis complex-2 gene expression. Int. J. Oncol..

[73] Zhang Y., Chao T., Li R., Liu W., Chen Y., Yan X., Gong Y., Yin B., Qiang B., Zhao J. (2008). MicroRNA-128 inhibits glioma cells proliferation by targeting transcription factor E2F3a. J. Mol. Med..

[74] Volovetz J., Berezovsky A.D., Alban T., Chen Y., Lauko A., Aranjuez G.F., Burtscher A., Shibuya K., Silver D.J., Peterson J. (2020). Identifying conserved molecular targets required for cell migration of glioblastoma cancer stem cells. Cell Death Dis..

[75] Shi G., Zhang Z. (2021). Rap2B promotes the proliferation and migration of human glioma cells via activation of the ERK pathway. Oncol. Lett..

[76] Li J., Zhu S., Kozono D., Ng K., Futalan D., Shen Y., Akers J.C., Steed T., Kushwaha D., Schlabach M. (2014). Genome-wide shRNA screen revealed integrated mitogenic signaling between dopamine receptor D2 (DRD2) and epidermal growth factor receptor (EGFR) in glioblastoma. Oncotarget.

[77] Sayyah J., Bartakova A., Nogal N., Quilliam L.A., Stupack D.G., Brown J.H. (2014). The Ras-related Protein, Rap1A, Mediates Thrombin-stimulated, Integrin-dependent Glioblastoma Cell Proliferation and Tumor Growth. J. Biol. Chem..

[78] Niola F., Zhao X., Singh D., Sullivan R., Castano A., Verrico A., Zoppoli P., Friedmann-Morvinski D., Sulman E., Barrett L. (2012). Mesenchymal high-grade glioma is maintained by the ID-RAP1 axis. J. Clin. Investig..

[79] Moon E.-Y., Lee G.-H., Lee M.-S., Kim H.-M., Lee J.-W. (2012). Phosphodiesterase inhibitors control A172 human glioblastoma cell death through cAMP-mediated activation of protein kinase A and Epac1/Rap1 pathways. Life Sci..

[80] She X., Yu Z., Cui Y., Lei Q., Wang Z., Xu G., Xiang J., Wu M., Li G. (2014). miR-128 and miR-149 enhance the chemosensitivity of temozolomide by Rap1B-mediated cytoskeletal remodeling in glioblastoma. Oncol. Rep..

[81] She X., Yu Z., Cui Y., Lei Q., Wang Z., Xu G., Luo Z., Li G., Wu M. (2014). miR-181 subunits enhance the chemosensitivity of temozolomide by Rap1B-mediated cytoskeleton remodeling in glioblastoma cells. Med. Oncol..

[82] Barrett A., Evans I.M., Frolov A., Britton G., Pellet-Many C., Yamaji M., Mehta V., Bandopadhyay R., Li N., Brandner S. (2014). A crucial role for DOK1 in PDGF-BB-stimulated glioma cell invasion through p130Cas and Rap1 signalling. J. Cell Sci..

[83] Lee H.K., Finniss S., Cazacu S., Xiang C., Poisson L.M., Blumberg P.M., Brodie C. (2015). RasGRP3 regulates the migration of glioma cells via interaction with Arp3. Oncotarget.

[84] Wang Q., Cai J., Fang C., Yang C., Zhou J., Tan Y., Wang Y., Li Y., Meng X., Zhao K. (2018). Mesenchymal glioblastoma constitutes a major ceRNA signature in the TGF-β pathway. Theranostics.

[85] Malchinkhuu E., Sato K., Maehama T., Yoshimoto Y., Mogi C., Kimura T., Kurose H., Tomura H. (2009). Role of Rap1B and Tumor Suppressor PTEN in the Negative Regulation of Lysophosphatidic Acid-induced Migration by Isoproterenol in Glioma Cells. Mol. Biol. Cell.

[86] Choudhury Y., Tay F.C., Lam D.H., Sandanaraj E., Tang C., Ang B.-T., Wang S. (2012). Attenuated adenosine-to-inosine editing of microRNA-376a* promotes invasiveness of glioblastoma cells. J. Clin. Investig..

[87] Li S., Xiang W., Tian J., Wang H., Hu S., Wang K., Chen L., Huang C., Zhou J. (2021). Bone Marrow-Derived Mesenchymal Stem Cells Differentially Affect Glioblastoma Cell Proliferation, Migration, and Invasion: A 2D-DIGE Proteomic Analysis. BioMed Res. Int..

[88] Jiang M., Zhuang Y., Zu W., Jiao L., Richard S.A., Zhang S. (2019). Overexpression of EPAC2 reduces the invasion of glioma cells via MMP-2. Oncol. Lett..

[89] Secord A.A., Teoh D.K., Barry W.T., Yu M., Broadwater G., Havrilesky L.J., Lee P.S., Berchuck A., Lancaster J., Wenham R.M. (2012). A Phase I Trial of Dasatinib, an Src-Family Kinase Inhibitor, in Combination with Paclitaxel and Carboplatin in Patients with Advanced or Recurrent Ovarian Cancer. Clin. Cancer Res..

[90] Miller A., Shah R., Pentsova E.I., Pourmaleki M., Briggs S., Distefano N., Zheng Y., Skakodub A., Mehta S.A., Campos C. (2019). Tracking tumour evolution in glioma through liquid biopsies of cerebrospinal fluid. Nat. Cell Biol..

[91] Saenz-Antoñanzas A., Auzmendi-Iriarte J., Carrasco-Garcia E., Moreno-Cugnon L., Ruiz I., Villanua J., Egaña L., Otaegui D., Samprón N., Matheu A. (2019). Liquid Biopsy in Glioblastoma: Opportunities, Applications and Challenges. Cancers.

[92] Bark J.M., Kulasinghe A., Chua B., Day B.W., Punyadeera C. (2020). Circulating biomarkers in patients with glioblastoma. Br. J. Cancer.

